# An integrated bioinformatics analysis to dissect kinase dependency in triple negative breast cancer

**DOI:** 10.1186/1471-2164-16-S12-S2

**Published:** 2015-12-09

**Authors:** Karen A Ryall, Jihye Kim, Peter J Klauck, Jimin Shin, Minjae Yoo, Anastasia Ionkina, Todd M Pitts, John J Tentler, Jennifer R Diamond, S Gail Eckhardt, Lynn E Heasley, Jaewoo Kang, Aik Choon Tan

**Affiliations:** 1 Division of Medical Oncology, Department of Medicine, School of Medicine, University of Colorado Anschutz Medical Campus, Aurora CO 80045 USA; 2 Department of Craniofacial Biology, School of Dental Medicine, University of Colorado Anschutz Medical Campus, Aurora CO 80045 USA; 3 Department of Computer Science, Korea University, Seoul, Korea; 4 Department of Biostatistics and Informatics, Colorado School of Public Health, University of Colorado Anschutz Medical Campus, Aurora CO 80045 USA

**Keywords:** Kinase dependency, Triple-Negative Breast Cancer, high-throughput screening, bioinformatics

## Abstract

**Background:**

Triple-Negative Breast Cancer (TNBC) is an aggressive disease with a poor prognosis. Clinically, TNBC patients have limited treatment options besides chemotherapy. The goal of this study was to determine the kinase dependency in TNBC cell lines and to predict compounds that could inhibit these kinases using integrative bioinformatics analysis.

**Results:**

We integrated publicly available gene expression data, high-throughput pharmacological profiling data, and quantitative *in vitro *kinase binding data to determine the kinase dependency in 12 TNBC cell lines. We employed Kinase Addiction Ranker (KAR), a novel bioinformatics approach, which integrated these data sources to dissect kinase dependency in TNBC cell lines. We then used the kinase dependency predicted by KAR for each TNBC cell line to query K-Map for compounds targeting these kinases. Wevalidated our predictions using published and new experimental data.

**Conclusions:**

In summary, we implemented an integrative bioinformatics analysis that determines kinase dependency in TNBC. Our analysis revealed candidate kinases as potential targets in TNBC for further pharmacological and biological studies.

## Background

Triple-negative breast cancer (TNBC) is a subtype of breast cancer that is lacking the expression ofestrogen receptor (ER), progesterone receptor (PR) and HER2 (ERBB2)[1]. TNBC, also known as basal-like breast cancer, is an aggressive disease with a poor prognosis. Unlike ER-positive, PR-positive, and HER2-amplified breast cancer subtype patients, chemotherapy is the only treatment option for TNBC patients. Advances in the treatment of TNBC have been hampered by the lack of novel effective targeted therapies due to the poor understanding of the underlying molecular characteristics of this disease. Recent large-scale molecular characterization studies in breast cancer have revealed some frequently mutated genes and altered pathways in TNBC[2,3]. These genes and pathways include *TP53*, *BRCA1/2*, *PIK3CA*, and *PTEN *mutations and activation of PI3K/AKT and RAS/RAF/MEK signaling pathways. Many of these genes and pathways are regulated by kinases (e.g. *PIK3CA*, *RAS*, *MAPKs*); therefore providing an opportunity to identify potential druggable targets by small moleculesfor TNBC therapy.

Protein kinases represent one of the largest “druggable” and well-studied protein families in the human genome[4]. This class of proteins (kinome) plays key role in regulating various signaling pathways in cells. There are>500 members of the human kinome which can be classified into seven different kinase families based on their conserved catalytic domain sequences[5]. In cancer cells, some kinases are mutated and have acquired oncogenic properties to drive tumorgenesis. Small molecules that inhibit these oncogenic kinases can effectively kill cancer cells. Targeted cancer therapies have exploited this “oncogene addiction” concept[6]; this has lead to several successful clinical applications of targeted therapies: BCR-ABL tyrosine kinase inhibition in chronic myeloid leukemia by imatinib[7], inhibition of *EGFR *in *EGFR*-mutated non-small cell lung cancers (NSCLC) by erlotinib or gefitinib[8-10], inhibition of *BRAF *in *BRAF*-mutated melanoma by vemurafenib[11]and inhibition of *ALK *in *EML4-ALK *NSCLC by crizotinib[12]. Furthermore, many of the small molecules inhibit multiple kinases and could be repositioned or repurposedfor other applications. For example, imatinib has been repositioned to inhibit *KIT *and *PDGFRA *in gastrointestinal stromal tumors[13] and crizotinib has been repositioned to inhibit *ROS1 *in *ROS1*-fusion NSCLC patients[14]. Large-scale quantitative *in vitro *kinase binding assays have been developed to capture the complex interactions between inhibitors and kinases[15-17].

High-throughput screening (HTS) provides a different perspective to interrogate biological systems using chemical biology. Large-scale HTS studies such as Cancer Cell Line Encyclopedia (CCLE)[18], Genomics of Drug Sensitivity in Cancer (GDSC)[19,20], Cancer Therapeutics Response Portal (CTRP)[21], and NCI-60 Developmental Therapeutic Program Screen[22]represent examples of the HTS pharmacological profiling data sources. One recent study has performed HTS of 180 kinase inhibitors in 12 TNBC cell lines[23]. Typically, HTS was performed on a panel of cancer cell lines screened with multiple compounds to generate pharmacological profiling data. From the pharmacological profiling data, one can correlate the compound sensitivity with other molecular genomics data to derive drug sensitivity signatures[18-21]. Another application of HTS pharmacological data is to correlate with *in vitro *kinase binding assays to deconvolute kinase dependency in biological systems[24]. However, no efforts have been made to integrate HTS pharmacological profiling data, *in vitro *kinase binding data, and genomics data for dissecting kinase dependency in cancer cells.

**Figure 1:**
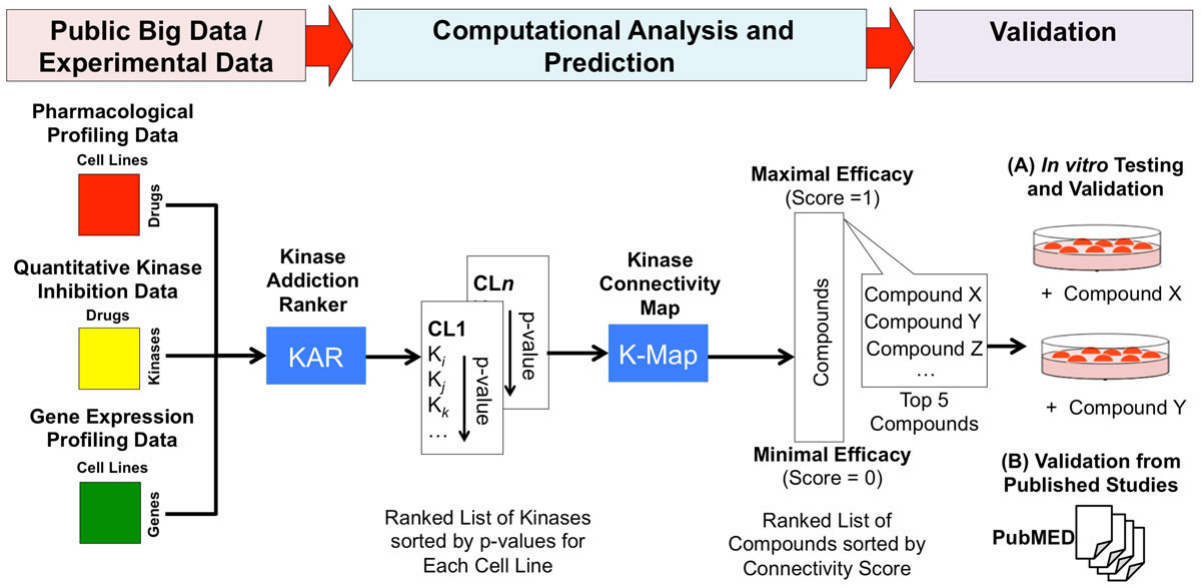
**Integrative bioinformatics research strategyto dissect kinase dependency in TNBC**. The Kinase Addiction Ranker (KAR) algorithm integrates gene expression, drug screen, and quantitative kinase-drug binding data to predict kinase dependence in TNBC cell lines. The top ranking kinases by KAR are then queried by K-Map to predict effective compounds. These predictions are then validated experimentally or in literature.

The goal of this study was to determine the kinase dependency in TNBC cell lines and to predict compounds that could inhibit these kinases using integrative bioinformatics analysis. In this study, we used publicly available gene expression data, HTS pharmacological profiling data, and quantitative *in vitro *kinase binding data. We employed our recently developed Kinase Addiction Ranker (KAR) to integrate these data sourcesto dissect kinase dependency in TNBC cell lines[25]. We then used the kinase dependency predicted by KAR to query K-Map [26,27]for connecting compounds with kinases for individual TNBC lines. For validation, we performed literature search on published experimental data and tested K-Map predictionsin cell lines. Our research strategy for this study is illustrated in Figure 1.

## Methods

### Pharmacological profiling data

We obtained the HTS pharmacological profiling data of 12 TNBC cell lines from a recently published paper [23]. The 12 TNBC cell lines are: BT20, BT549, CAL148, HCC38, HCC70, HCC1143, HCC1187, HCC1806, Hs578T, MDA-MB231, MDA-MB468 and MFM223. These cell lines were screened with 180 kinase inhibitors. The drug sensitivity read out from this dataset is half maximal effective concentration values (EC_50_).

### Quantitative kinase inhibition data

We obtained comprehensive quantitative kinase inhibition data for 72 of the 180 screened kinase inhibitors from literature. Drug sensitivity data from these 72 drugs were used in our algorithm. This comprehensive inhibition data allows for better interpretation of high-throughput screening results as most kinase inhibitors interact with far more kinases than the ones that are most commonly reported[15].

### Microarray gene expression data

We obtained the TNBC microarray gene expression data from the Cancer Cell Line Encyclopedia (GSE36133). These cell lines were profiled using Affymetrix HG-U133 Plus 2.0 microarrays. Raw CEL files for these cell lines were normalized using Robust Multiarray Average (RMA) approach in Affymetrix Power Tools (APT).

### Kinase Addiction Ranker (KAR)

We have recently developed KAR (Kinase Addiction Ranker), a novel computational method that integrates high-throughput drug screening data, quantitative kinase binding data, and transcriptomics data to define kinase dependency for individual cancer cell lines [25]. For each cell line, KAR first assigns compounds in the high-throughput drug screen to1 of 5 bins based on drug sensitivity. The bin number determines how many points each kinase target of the drug receives by the scoring algorithm. Targets of compounds in Bin 1 receive 20 points, Bin 2 targets receive 10 points, Bin 3 targets receive 5 points, Bin 4 targets receive 0 points, and Bin 5 targets receive -10 points. Bin 4 and 5 therefore contain drugs that do not meet the threshold for drug sensitivity in the sample, with compounds in Bin 5 receiving a negative penalty.

Next, quantitative kinase binding data is dichotomized as inhibited or not inhibited for each compound based on user-defined threshold (default: a kinase is considered as inhibited by the compound if IC_50_/K_d_< 1 μM or percent of inhibition > 85%). Transcriptomics data is used to filter out low expressed kinases. Kinases are scored by adding or subtracting points based on the sensitivity bin of each drug that inhibits the kinase. Finally, p-values are computed using chi-square and Fisher's exact tests to determine if there is a significant association between a kinase being inhibited and the drug being sensitive (Sensitivity Bins 1-3) in the cell line. KAR returns the ranked list of kinases based on p-values and scaled scores. Kinases with p < 0.05 will be deemed as significant and dependent by the cancer cell line. We have implemented KAR in two programming languages: python and Matlab. KAR is freely available at: http://tanlab.ucdenver.edu/KAR.

### Kinase Connectivity Map (K-Map) Analysis

We recently developed and implemented K-Map that systematically connects a kinase profile with a reference kinase inhibitor database and predicts the most effective inhibitor for a queried kinase profile [26,27]. The K-Map consists of three key components: (1) a reference database that contains a set of kinase inhibitors profiles; (2) a query signature; and (3) a pattern matching algorithm or similarity metric defined between a query signature and a reference kinase inhibitor profile to quantify the connection (or similarity) between the interactions of kinases and inhibitors.

#### Reference Database

The current K-Map reference database was builtbased on two recently published comprehensive analyses of kinase inhibitor selectivity [15,16]. The first study systematically interrogated 178 commercially available inhibitors against a panel of 300 protein kinases using a radiometric phospho-transfer method to assess the percent kinase inhibition (IC_50_)[15]. The second study measured the selectivity and potency of 72 inhibitors against 442 kinases using direct binding affinities between inhibitors and kinases (K_d_)[16]. These datasets were converted into rank-ordered lists according to the inhibitors' potencies against the kinases and used as the K-Map reference profiles for matching query kinases.

#### Query Signature

For each TNBC line, the top five kinases ranked by KAR were used as the query kinase profile and connected through the K-Map in this study.

#### Pattern Matching Algorithm

K-Map implementsthe pattern matching strategy based on the Kolmogorov-Smirnov (KS) statistics. The KS-test is a non-parametric, rank-based pattern-matching approach implemented in the connectivity map[28]. The algorithm aims to correlate kinase inhibitors, based on kinase inhibition profiles in the reference database, with a given query (i.e. top five kinases ranked by KAR). For every inhibitor in the reference database, the KS statistic is computed and a “connectivity score” is defined where it ranges from 1 (maximal efficacy) to 0 (minimal efficacy). K-Map then returnsthe ranked list of kinase inhibitors that best inhibit the list of queried kinases sorted by their “connectivity scores”. We used K-Map to connect the top five kinases for 12 TNBC cell lines with drugs in this study. K-Map is freely available at: http://tanlab.ucdenver.edu/kMap.

### Cell lines and culture

HCC1806 was obtained from the American Type Culture Collection (ATCC). The cell line has been authenticated as previously described[29,30]. Cell cultures were prepared as previously described[29,30].

### Drugs

Erlotinib and bosutinib were obtained commercially (Selleck Chemicals) and prepared according to the manufacture's guidelines.

### Cell proliferation assay

To evaluate the drug effects in TNBC cells, we used the CellTiter-Glo assay. In brief, cell viability assayswere performed using CellTiter-Glo (Promega) according to manufacturer's instructions. TNBC cells were seeded at 4000 cells/well in a 96-well plate, and exposed to increasing concentrations of erlotinib or bosutinib from 0 - 10 μmol/L for 96 hours. CellTiter-Glo measurements were obtained for these different concentrations to determine cell viability. Cell viability curves were derived from the data and IC_50 _values calculated from a minimum of three experiments.

## Results and discussion

### Kinase Addiction Ranker for ranking kinase dependency in TNBC cell lines

To identify kinase dependency in TNBC, we first analyzed a HTS pharmacological profiling data set of 180 kinase inhibitors profiled across 12 TNBC cell lines[23]. We selected 72 of the 180 profiled drugs based on availability of a published quantitative *in vitro*kinase inhibition profile and inhibition of at least one kinase above threshold (K_d_/EC_50_<1 μM or >85% inhibition). We used KAR (Kinase Addiction Ranker), a novel bioinformatics approach, which integrates gene expression, drug sensitivity, and kinase inhibition data to generate a ranked list of kinase dependency in these TNBC cell lines. As described in the methods section, KAR integrates three data sources (pharmacological profiling data, kinase inhibition data and gene expression data) to delineate kinase dependency in individual cell lines. On average, KAR identified 24 kinases with a significant association with drug sensitivity in each cell line (range: 9 - 46) (Table 1).

**Table 1:** Top 5 kinases ranked by KAR for the 12 TNBC lines.

TNBC Cell Line	Top 5 Kinases (Score, p-value) ranked by KAR				
	
(Number of significant kinases, p < 0.05)	Rank 1	Rank 2	Rank 3	Rank 4	Rank 5
BT20	*EGFR*	*MAP4K2*	*MAP4K3*	*MAP3K9*	*MARK4*
(9)	(160, 0.0095)	(150, 0.0095)	(155, 0.016)	(160, 0.0257)	(90, 0.039)
BT549	*MAP4K4*	*MINK1*	*MAP3K11*	*MAP4K5*	*NUAK1*
(42)	(115, 1.1 × 10^-5^)	(80, 7.1 × 10^-5^)	(95, 0.0001)	(120, 0.0002)	(110, 0.0002)

CAL148	*MAP4K2*	*CDK5 */ p25	*CDK2 */ cyclin A	*IRAK1*	*AURKB*
(14)	(75, 0.0029)	(65, 0.0073)	(60, 0.0073)	(40, 0.0084)	(95, 0.0108)

HCC38	*MAP3K9*	*MAP4K5*	*MARK4*	*MAP4K4*	*MINK1*
(43)	(85, 1.79 × 10^-5^)	(95, 5.73 × 10^-5^)	(85, 5.73 × 10^-5^)	(100, 8.05 × 10^-5^)	(85, 9,47 × 10^-5^)

HCC70	*PRKD3*	*CDK5 */ p25	*STK3*	*MAP3K9*	*AURKA*
(24)	(90, 0.0015)	(100, 0.0031)	(75, 0.0115)	(65, 0.0127)	(45, 0.0129)

HCC1143	*CDK5 */ p25	*MST4*	*CDK1*/cyclin A	*CDK3*/cyclin E	*RAF1*
(46)	(45, 0.0005)	(40, 0.0005)	(30, 0.0041)	(30, 0.0041)	(10, 0.0041)

HCC1187	*CLK4*	*ROCK2*	*PIK3CA*	*GSK3B*	*MAP3K9*
(13)	(60, 0.0182)	(50, 0.0182)	(45, 0.0182)	(85, 0.0197)	(85, 0.0229)

HCC1806	*YES1*	*TNK2*	*EGFR*	*MAP4K4*	*LYN*
(23)	(195, 0.0004)	(180, 0.0004)	(125, 0.0041)	(170, 0.0058)	(130, 0.0072)

HS578T	*PDGFRB*	*MAP4K4*	*LYN*	*MTOR*	*CLK4*
(22)	(60, 0.0005)	(50, 0.0022)	(70, 0.0024)	(60, 0.0024)	(30, 0.0037)

MDA-MB-231	*MAP3K7*	*CDK1 */ cyclin A	*IGF1R*	*CDK5 */ p25	*CDK6 */ cyclin D1
(32)	(40, 0.0007)	(40, 0.0007)	(25, 0.0007)	(35, 0.0013)	(25, 0.0039)

MDA-MB-468	*CDK2 */ cyclin A	*CDK5 */ p25	*MAP3K9*	*STK4*	*PRKD3*
(10)	(95, 0.0047)	(95, 0.0047)	(155, 0.0062)	(155, 0.0062)	(90, 0.0164)

MFM-223	*ROCK2*	*CLK4*	*AURKA*	*RET*	*MAP3K9*
(13)	(50, 0.0043)	(45, 0.0043)	(35, 0.0043)	(40, 0.0126)	(25, 0.0171)

The kinases most commonly associated with drug sensitivity in the TNBC cell lines were *MAP4K4 *and *PRKD3*, which were each significant in 10/12 TNBC cell lines. *MAP4K4 *(also known as HGK or NIK) activates *MAPK8*/JNK signaling. *MAP4K4 *is involved in cell migration and invasion in melanoma, ovarian, breast and prostate cancers[31]. Moreover, overexpression of *MAP4K4 *correlated with larger tumor size, increased lymph node involvement, and recurrence in pancreatic ductal carcinoma[32]. *PRKD3 *has been shown to promote proliferation and chemoresistance in TNBC [33,34]. Since *MAP4K4 *and *PRKD3 *are so frequently associated with drug sensitivity in this dataset, they may represent targets that could benefit larger populations of TNBC patients. *MAP4K4 *and *PRKD3 *are simultaneously inhibited by CDK1/2 inhibitor III and PKR inhibitor, which were sensitive in nearly all of the 12 cell lines.

**Figure 2:**
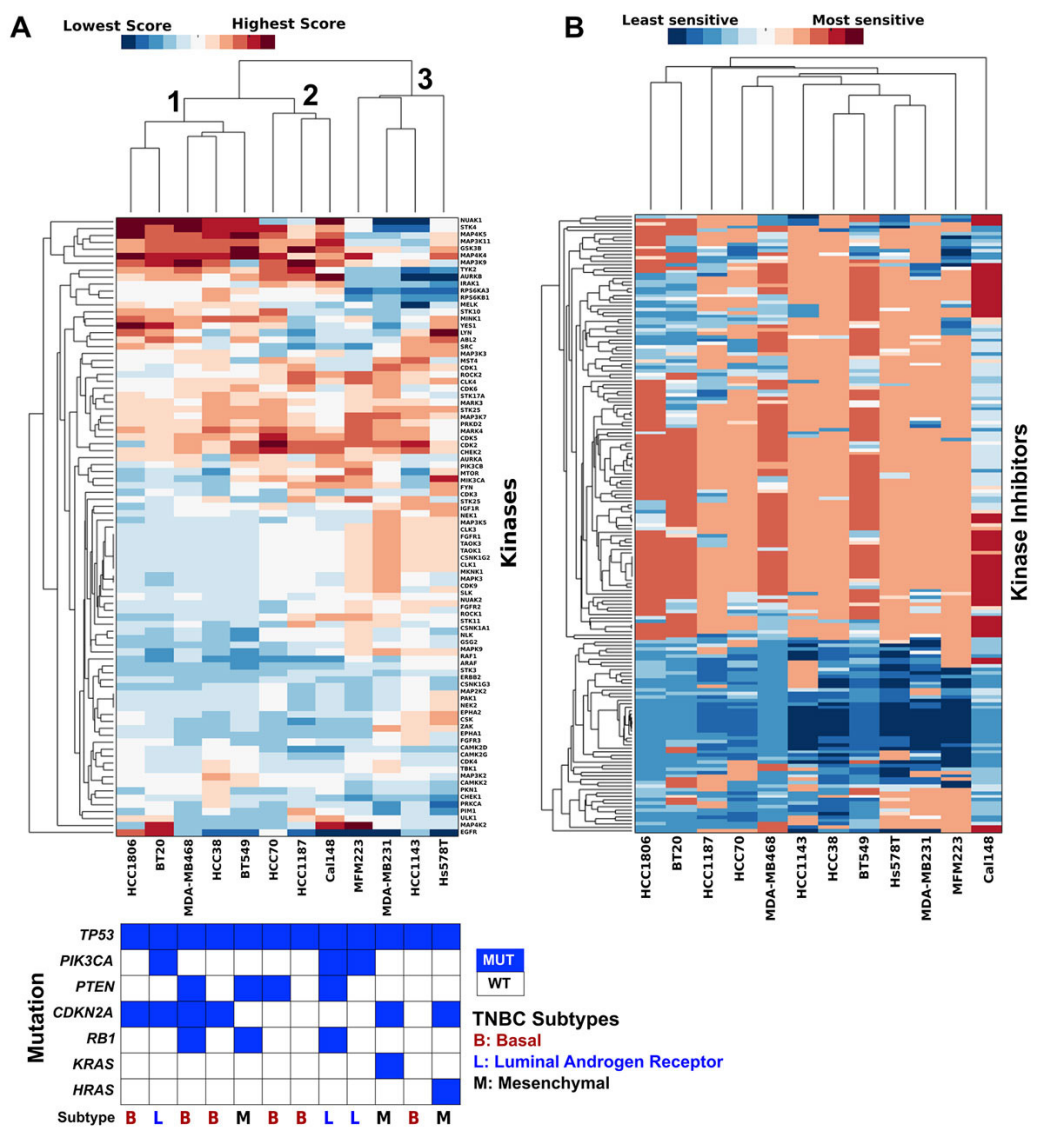
**KAR identifies relationships in kinase dependency in TNBC**. **A**. Hierarchical clustering ofTNBC cell lines and kinases based on scaled kinase dependency scores. Each column was normalized to give a mean of 0 and a standard definition of 1. Red indicates that a cell line has a high dependence on a given kinase and blue indicates low dependence. Mutation and subtype information are also provided [1]. **B**. Hierarchical clustering of TNBC cell lines based on kinase inhibitor sensitivity data (from [23]). Clustering based on kinase inhibitor sensitivity resulted in different groupings compared to kinase dependency score. Red indicates that a cell line has higher sensitivity to a particular kinase inhibitor (lower IC_50_) and blue indicates lower sensitivity (higher IC_50_).

Next, we performed hierarchical clustering on the scaled KAR scores to reveal relationships between the 12 TNBC cell lines and kinases (Figure 2). Clustering included the 89 kinases that had a significant association with drug sensitivity in at least one of the 12 cell lines analyzed. No correlation of TNBC subtypes[1] were found in these clusters, this is similar to the previous published pharmacological profiling data. This suggests the heterogeneity of the molecular subtypes of TNBC andthat understanding the kinase dependency could provide better treatment strategy for this disease.

From Figure 2, the cluster analysis reveals three main groups of TNBC cell lines (Figure 2A). The first group contains HCC1806, BT20, MDA-MB-468, HCC38 and BT549, the second group contains HCC70, HCC1187 and CAL148 and the third group contains MFM223, MDA-MB-231, HCC1143 and Hs578T. Within the first group, HCC1806 and BT20 show a unique dependence on EGFR when compared to the other three lines (MDA-MB-468, HCC38 and BT549). Interestingly, EGFR also does not cluster with any of the other kinases analyzed, indicating EGFR dependence is a fairly unique marker in a cell line compared to other kinases.

We also clustered the pharmacological profiling data (EC_50_) from all 180 drugs[23](Figure 2B). As with the kinase score, *EGFR*-dependent BT20 and HCC1806 grouped together. The other cell lines show less distinct groupings with many cell lines being paired with different cell lines than when clustered based on kinase score. This suggests that the kinase dependency relationships derived from KAR are different from the relationships derived from clustering pharmacological profiling data.

### Validating kinase dependency in TNBC cell lines

Here, we validate the kinases with high KAR rankings (Table 1) in a subset of the TNBC cell lines studied based on previously published studies.

#### BT20

KAR ranks Epidermal Growth Factor Receptor (*EGFR*), as the top kinase for this cell line. Indeed, previously published papers have verified that this cell line expressed high levels of *EGFR*[35,36], however, this cell line is not sensitive to EGFR inhibitors such as erlotinib or gefitinib[35-37]. This indicates that there may be some other kinases driving the proliferation of this cell line.

#### HCC1806

For this cell line, KAR ranks *YES1 *and *LYN *in the top 5 kinases. Both of these kinases are *SRC *kinase family members. Indeed, previous studies have demonstrated that this cell line is highly sensitive to dasatinib (FDA approved *SRC *inhibitor)[38]. Interestingly, KAR also ranks *EGFR *as one of the kinase dependent in this cell line. From the clustering of kinase dependency score (Figure 2), BT20 and HCC1806 clustered together.

#### HCC70

KAR ranks Aurora Kinase A (*AURKA*) as one of the top five kinases in this cell line. Previously, we have tested two different Aurora Kinase inhibitors across a large panel of TNBC cell lines, and found that HCC70 is very sensitive to MLN8237 (IC_50 _= 0.1 μM)[30] and ENMD2076 (IC_50 _= 0.549 μM)[29]. This supports *AURKA *dependence in this cell line. In fact, there is an ongoing Phase II clinical trial of treating TNBC patients with ENMD2076 (http://ClinicalTrials.gov ID: NCT01639248).

#### MDA-MB-231

KAR ranks *MAP3K7*, which is commonly known as the Transforming growth factor beta-activated kinase 1 (*TAK1*), as the top kinase for this cell line. Interestingly, this cell line is the only TNBC cell line analyzed that is a *KRAS *mutant (*p.G13D*) and is highly dependent on *KRAS *("*KRAS*-dependent" cell line)[39]. Indeed, previous studies have demonstrated that this cell line has high *MAP3K7 *expression, and is sensitive to the *TAK1 *kinase inhibitor 5Z-7-oxozeaenol [40]. Previous studies in colorectal cancer cell lines have suggested that *TAK1 *could be a therapeutic target in *KRAS*-dependent lines [41]. This confirms that KAR could identify relevant kinases for individual cell lines.

KAR results indicate that the other TNBC cell lines seem highly dependent on MAPKs (e.g. *MAP4K2*, *MAP4K3*) and CDK kinases (e.g. *CDK1*, *CDK2*, *CDK3*, *CDK5*, *CDK6*). A previous study evaluating kinase expression in Estrogen Receptor (ER) positive vs. negative breast cancer samples identified a subgroup in the ER-negative samples also enriched with MAPKs[42].

### Predicting compounds for individual TNBC cell lines by using K-Map

For each TNBC cell lines, we used the top five ranking kinases (lowest chi-square p-values) as the query to K-Map for predicting effective compounds. Compounds with p < 0.05 are selected and sorted by connectivity scores. Table 2 lists the top five compounds predicted by K-map based on the top five kinases for each TNBC cell lines.

**Table 2:** To 5 kinase inhibitors predicted by K-Map for the 12 TNBC lines.

	Top 5 Drugs (Score) ranked by K-Map				
	
TNBC Cell Line	Rank 1	Rank 2	Rank 3	Rank 4	Rank 5
BT20	Staurosporine	Bosutinib	Go 6976	TWS119	PKR Inhibitor
	(1.00)	(0.976)	(0.972)	(0.963)	(0.953)

BT549	Staurosporine	K-252a	SB 218078	CDK1/2 Inhibitor III	Go 6976
	(1.00)	(1.00)	(0.997)	(0.995)	(0.995)

CAL148	Lestaurtinib	K-252a	Staurosporine	JAK3 Inhibitor VI	CDK1/2 Inhibitor III
	(1.00)	(1.00)	(1.00)	(0.996)	(0.994)

HCC38	Staurosporine	K-252a	CDK1/2 Inhibitor III	SB 218078	Go 6976
	(1.00)	(1.00)	(0.995)	(0.991)	(0.989)

HCC70	Lestaurtinib	Staurosporine	CDK1/2 Inhibitor III	K-252a	SB 218078
	(1.00)	(1.00)	(0.998)	(0.998)	(0.983)

HCC1143	JNK Inhibitor II	CDK4 Inhibitor III	CDK4 Inhibitor II	VEGFR Receptor Inhibitor II	CHK2 Inhibitor II
	(0.977)	(0.943)	(0.938)	(0.936)	(0.929)

HCC1187	Staurosporine	CDK1/2 Inhibitor III	JAK3 Inhibitor VI	JNJ-7706621	PKR Inhibitor
	(1.00)	(0.998)	(0.990)	(0.973)	(0.971)

HCC1806	Bosutinib	TWS119	Staurosporine	Dasatinib	WHI-P154
	(1.00)	(0.990)	(0.98)	(0.966)	(0.956)

HS578T	Staurosporine	SU11652	K-252a	Sunitinib	Dorsomorphin
	(1.00)	(0.992)	(0.986)	(0.984)	(0.982)

MDA-MB-231	CDK1/2 Inhibitor III	Indirubin Derivative E804	Sunitinib	Aminopurvalanol A	CDK2 Inhibitor IV
	(1.00)	(0.975)	(0.956)	(0.949)	(0.94)

MDA-MB-468	Lestaurtinib	Staurosporine	CDK1/2 Inhibitor III	K-252a	JAK3 Inhibitor VI
	(1.00)	(1.00)	(0.998)	(0.998)	(0.99)

MFM-223	Staurosporine	CDK1/2 Inhibitor III	K-252a	JAK3 Inhibitor VI	SYK Inhibitor
	(1.00)	(1.00)	(0.99)	(0.974)	(0.972)

Staurosporine, a multi-kinase inhibitor used as the positive control in the K-Map, was predicted as an effective compound for ten TNBC cell lines (Table 2). This is likely because a highly non-specific compound like Staurosporine can inhibit > 400 kinases by itself.

### Validating compounds predicted by K-Map in TNBC cell lines

**Figure 3:**
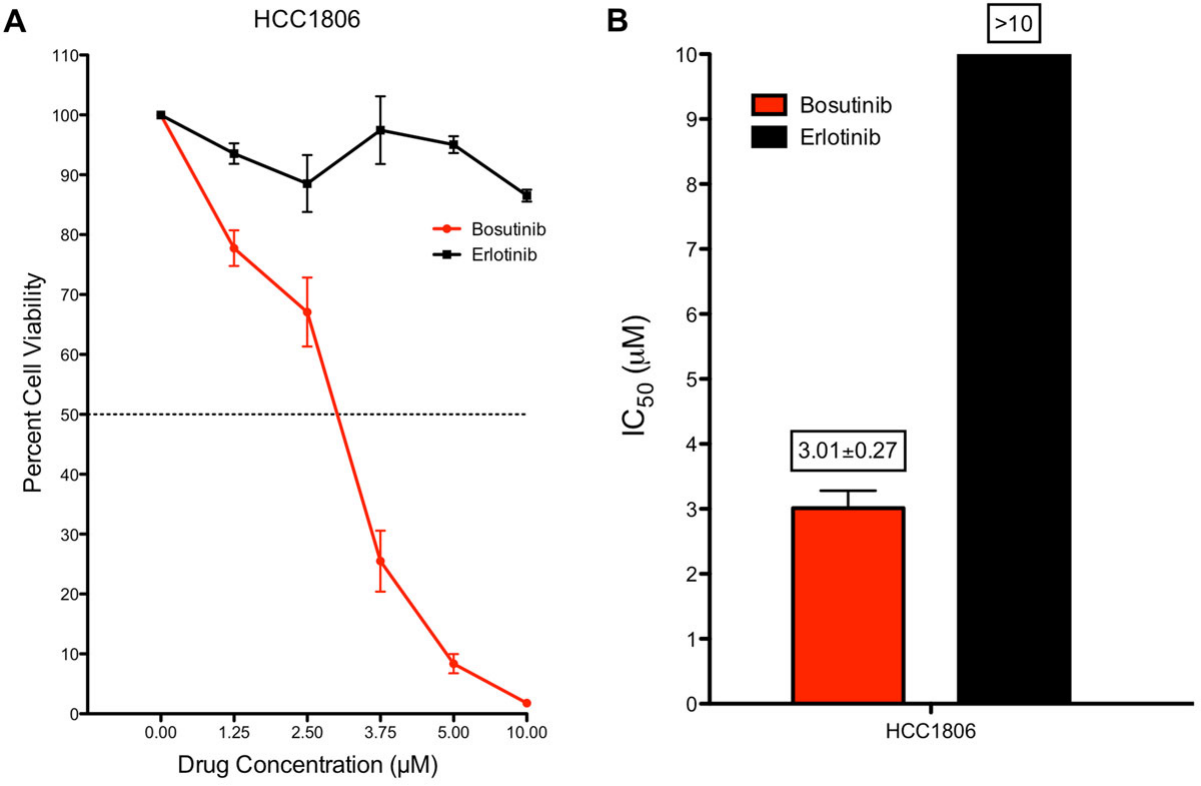
**Validation of bosutinib and erlotinib in TNBC cell line HCC1806**. **A**. Cell viability (mean +/- SE) dose response for bosutinib and erlotinib in HCC1806. **B**. Estimation of the IC_50 _for bosutinib and erlotinib in HCC1806. HCC1806 was much more sensitive to bosutinib than erlotinib. While both compounds target EGFR (KAR rank = 3), bosutinib targets other top ranking kinases by KAR: *YES1*, *TNK2*, *MAP4K4*, and *LYN*.

From Table 2 K-Map predicts bosutinib as one of the compounds that targets the top five ranking kinases of BT20 and HCC1806. Both cell lines have *EGFR *dependency as determined by KAR, and one of the targets of bosutinib is EGFR. In addition to *EGFR*, bosutinib also inhibits top ranking kinases *YES1*, *TNK2*, *MAP4K4*, and *LYN *(Table 1). Therefore, bosutinib inhibits each top ranking kinase for HCC1806 while erlotinib only inhibits one of the top five (*EGFR*). We would therefore predict that HCC1806 would be more sensitive to bosutinib than erlotinib. To validate this prediction, we tested HCC1806 *in vitro *with bosutinib and erlotinib using a CellTiter-Glo assay. As depicted in Figure 3, the IC_50 _of bosutinib(3 μM) is lower than erlotinib (>10 μM) in HCC1806. This validates the K-Map prediction that this cell line is more sensitive to bosutinibthan erlotinib. We also validated BT20 with bosutinib and erlotinib, and found that bosutinib also exhibited lower IC_50 _than erlotinib. (data not shown).

K-Map predicts SU11652 and sunitinib as potential compounds to be effective against Hs578T. Both compounds are *PDGFR *inhibitors, where *PDGFRB *is the top dependent kinase predicted by KAR for this cell line. Indeed, previous studies have demonstrated that Hs578T has high expression of *PDGFRB *(both at mRNA and protein levels), and this cell line is more sensitive to sunitinib[43].

CDK 1/2 inhibitor was predicted by K-Map as one of the compounds that inhibits the top five kinases in nine TNBC cell lines (BT549, CAL148, HCC38, HCC70, HCC1143, HCC1187, MDA-MB-231, MDA-MB-468 and MFM-223). Recent studies have suggested that *MYC*-dependence is synthetic lethal with CDK inhibitor in TNBC cell lines[44]. Indeed, six of these cell lines (BT549, HCC38, HCC70, HCC1143, MDA-MB-231 and MDA-MB-468) were *MYC*-dependent [45]. This supports that the K-Map prediction of CDK1/2 inhibitor could be a potential therapeutic for these TNBC cell lines.

Similar to Fink et al.'s analysis of the pharmacological profiling data [23], we observed heterogeneity of kinase dependence among the 12 TNBC cell lines and no co-clustering of cell lines of the same molecular subtype. We also showed *EGFR *dependence for BT-20 and HCC1806, but our experiments showed much lower sensitivity to Erlotinibthan Fink et al. (IC_50 _0.2 μM, our experiments: >10 μM). Fink et al.'s clustering of the drug sensitivity datarevealed co-clustering of HCC70, BT549, and MDA-MD468, and reported increased sensitivity of this group to PI3K pathway inhibitors[23]. Here, KAR revealed significant association between *PIK3CB *inhibition and drug sensitivity in HCC70 and BT549, but much higher correlations with drug sensitivity for other kinases (Table 1). Fink et al. also report that another group of cell lines which co-cluster (HCC38, HCC1143, HCC1187, HS578T, MDA-MB231, and MFM-223), are generally resistant to kinase inhibition with no kinase target being selectively toxic to this group [23]. Our approach incorporating more comprehensive target lists for each drug, however, was able to find kinases with significant associations with drug sensitivity for each cell line in this group. Moreover, *MAPK4K4*, which was one of the kinases most commonly associated with drug sensitivity in the 12 TNBC cell lines, is significant in all but HCC1187in this group of cell lines.

Here, we presented examples of how the KAR algorithm and K-Map research tool can be integrated to determine kinase dependency and predict effective cancer drugs for TNBC. KAR aids greatly in preventing misinterpretation of HTS data, as kinase inhibitors typically inhibit many more targets than are commonly reported. KAR therefore helps uncover kinase dependency that may be overlooked if only focusing on the commonly reported drug targets. Moreover, incorporation of gene expression data can help ensure that high-ranking kinases will be translationally applicable. Compared to previous approaches[24,46-48], KAR benefits from producing scores and p-values that can be easily interpreted by biologists without computational backgrounds, incorporation of transcriptomics data, increased accessibility (MATLAB and python functions available at http://tanlab.ucdenver.edu/KAR), and does not require preliminary optimization of the drug screening list. K-Map allows for quick connection of essential kinases revealed by KAR to compounds for experimental testing. K-Map can help reveal drugs that may not have been part of the original screening set. While we used this approach with TNBC cell lines, a similar strategy can be used with patient samples to predict effective kinase inhibitor therapies and drug combinations.

## Conclusions

We presented an integrative bioinformatics analysis to determine kinase dependency in TNBC. We integrated three different high-throughput data sources with the KAR algorithm: HTS pharmacological profiling data, quantitative *in vitro *kinase binding data, and gene expression data. We then queried the top five kinases from each TNBC cell lines to K-Map to predict compounds that could inhibit these sets of kinases. We validated the KAR and K-Map predictions using experiments and published studies. Using the integrative bioinformatics analysis, we have discovered kinase dependency in these TNBC cell lines. The data provide candidate kinases and drugs for further pharmacological and biological studies.

## List of abbreviations used

AURKA: Aurora Kinase A; EGFR: Epidermal Growth Factor Receptor; HTS: High-throughput Screening; KAR: Kinase Addiction Ranker; NSCLC: Non-small cell lung cancer; TNBC: Triple Negative Breast Cancer.

## Competing interests

The authors declare that they have no competing interests.

## Authors' contributions

KAR and ACT conceived the study, coordinated the experiments, performed acquisition of data, participated in data analysis, and drafted the manuscript. LEH and JK contributed to the study design. KAR, JK, JS, MY and ACT conducted bioinformatics analysis. PK, AI, TMP, JTT, JRD and SGE conducted experimental validation. All authors read and approved the final manuscript.

## Declaration

Publication charges for this article have been funded bythe David F. and Margaret T. Grohne Family Foundation.

This article has been published as part of *BMC Genomics *Volume 16 Supplement 12, 2015: Joint 26th Genome Informatics Workshop and 14th International Conference on Bioinformatics: Genomics. The full contents of the supplement are available online at http://www.biomedcentral.com/bmcgenomics/supplements/16/S12.
